# Compositional Reasoning in Early Childhood

**DOI:** 10.1371/journal.pone.0147734

**Published:** 2016-09-02

**Authors:** Steven Piantadosi, Richard Aslin

**Affiliations:** Department of Brain and Cognitive Sciences, University of Rochester, Rochester, NY, United States of America; University of Kansas Medical Center, UNITED STATES

## Abstract

Compositional “language of thought” models have recently been proposed to account for a wide range of children’s conceptual and linguistic learning. The present work aims to evaluate one of the most basic assumptions of these models: children should have an ability to represent and compose *functions*. We show that 3.5–4.5 year olds are able to predictively compose two novel functions at significantly above chance levels, even without any explicit training or feedback on the composition itself. We take this as evidence that children at this age possess some capacity for compositionality, consistent with models that make this ability explicit, and providing an empirical challenge to those that do not.

## 1 Introduction

One of the basic goals of cognitive science is to understand the nature of conceptual representations. Proposals for the format and structure of concepts range through theories based on prototypes [1], exemplars [2, 3], language-like representations [4], and others [5]. A central issue for all approaches is that of *compositionality*—how to handle the combination of individual concepts into more complex representations (e.g. “beautiful” composed with “accordions” yields a composite concept, “beautiful accordions”) [6–9]. This ability may be built “on top” of a representational format like prototypes [10] or it may be intrinsically a part of the core conceptual representation itself, as is hypothesized in *language of thought* (LOT) theories. According to LOT theories, the formation of a complex thought like “all sailboats are heavy” occurs when thinkers construct the corresponding compositional structure built out of simpler operations. For instance, such a conceptual format may be similar to standard logic: ∀*x*.*sailboat*(*x*)→*heavy*(*x*). Here, ∀, *sailboat* and *heavy* are functions that are composed in one particular way to express the composite idea “all sailboats are heavy.” These functions may themselves be built out of simpler operations, but in LOT theories eventually this process grounds out in primitive functions from which all other thoughts and representations are derived.

LOT theories are motivated in part by the rich structure human concepts appear to easily represent, as well as by factors like the systematicity, productivity, and compositionality of cognition [11]. Indeed, the LOT has a rich history in cognitive science, dating back at least to Boole [12] and later Frege [13]. Boole sought to characterize the “laws of thought” and did so by postulating the formal logical system of Boolean logic, which builds logical representations (formulas) out of the logical primitives *and*, *or*, and *not*. More recent incarnations of LOT theories have argued specifically for compositional, symbolic representations as fuller cognitive theories encompassing a wider range of semantic and logical operations [4]. A structured LOT has also been argued to explain developmental change in concept and language learning [14–21], providing a convenient formalism to express both what learners bring to learning tasks (primitive functions), and what exactly they acquire (compositions of those functions to explain observed data). For instance, Piantadosi et al. [21] suggest that children may know about simple functions on sets (e.g. union, intersection, etc.) and build more complex representations of cardinality and counting by appropriately composing these operations. This type of general approach builds on work of Feldman [22] who used a compositional system to explain patterns in Boolean concept learning, and Siskind [14] who first developed a compositional account of learning semantics. Following Goodman et al. [16], many recent theories posit rational (Bayesian) statistical models as the core *inductive* mechanism that decides between possible combinations of primitives in the face of data. In all cases—dating back to Boole—the core claim is that composition of simple primitive functions is what allows for the creation of complex cognitive representations.

While learning studies and modeling work have established LOT-based models as plausible *in principle*, no experimental work has examined LOT models’ most basic assumptions: learners should be able to represent functions and combine them through composition. This ability should be present without any explicit instruction on either the fact that functions can be composed, or what happens when they are composed. This ability is not trivial. Functions may perform many kinds of operations and in principle function combination can be used to express arbitrary computation [23–25].

To be clear, we focus here on *conceptual* compositionality (combinations of concepts), not *linguistic* compositionality (combinations of words in sentences), which has been studied previously [26–29]. The reason for this is that acquisition models have so far focused on learning either individual word meanings by composing simpler conceptual elements [14] or using compositional LOT systems to learn non-linguistic systems of concepts like magnetism [19]. In these cases, the learning models therefore make the strong prediction that children should be able to compose operations outside of the domain of language. Note that an ability to compose linguistic expressions does not establish that children will be able to compose conceptual operations; the compositionality of linguistics may or may not be encapsulated within language processing. The present work takes a first step towards investigating non-linguistic conceptual combination in children.

Of course, it is important to note that many tasks can be *interpreted* as compositional, depending on how they are formalized. Even, for instance, Sally-Ann [30] or simple numerical tasks with infants [31] require multiple updates to a representation, and therefore can be phrased as compositions of functions. Here, however, we approach compositionality from a more directed stance, constructing an experiment that is difficult to interpret in any way but compositional. The general logic of our experiment is to teach children that certain “screens” (occluders) cause specific feature changes (color or pattern) to an occluded object. The critical test conditions occur when two such screens are adjacent and children observe an object go behind both screens but they do not observe it between screens. Prediction of the correct outcome in this case requires them to apply both object transformations. Children receive no feedback on their responses to the compositional (two-screen) trials, meaning that success is indicative of *automatic* compositional reasoning without instruction or encouragement.

This experiment tests whether children can compute a composition of functions like *f*_2_(*f*_1_(*x*)), where *f*_2_ is the second screen and *f*_1_ is the first. However, there is a stronger sense in which learners might “know” compositionality: they might be able to form an explicit symbol *h* = *f*_2_(*f*_1_(*x*)) for the composition itself. Creating such a symbol is not a necessary ability for success on our task. In a linguistic analogy, the strong form of compositionality is tantamount to knowing that the term (symbol) “pit stop” refers to “refueling” and “changing tires.” The weaker form corresponds to being able to refuel and then change tires, without knowing that there is a term or symbol that refers to the combination of both operations. Because we are just beginning to explore the type of compositional reasoning in a non-linguistic task, we start by investigating the developmental origins of the weaker sense: can preschoolers predictively compose novel operations? Or, do they show catastrophic failures when attempting to combine multiple functions, perhaps reminiscent of younger children’s limitations with multiple updates to representations of objects or numerosity [32–34]?

## 2 Methods

**Fig 1:**
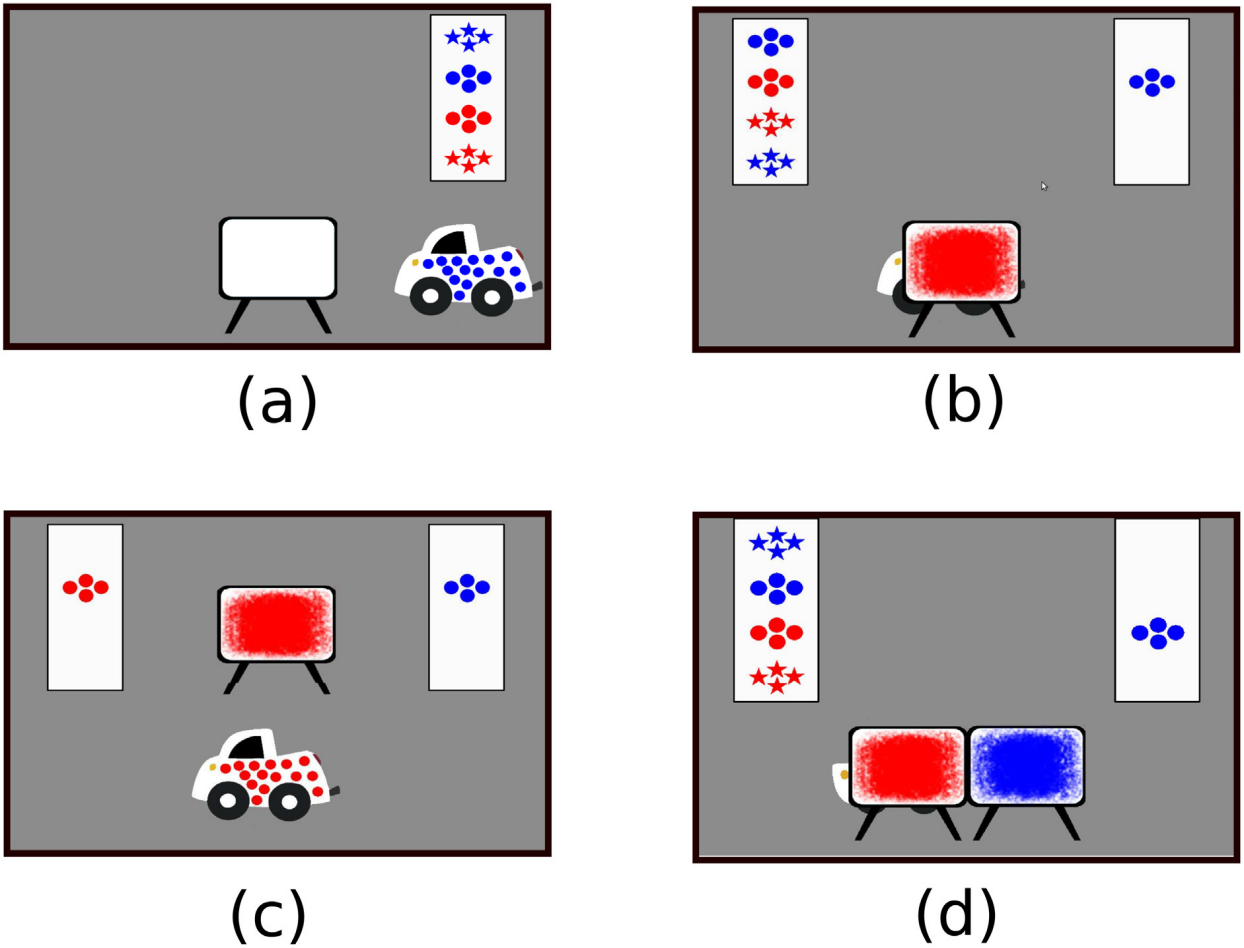
The four phases of the experiment. First, a truck is observed (a) and children are required to touch which pattern of the four possible patterns in the box matches the car. The response of the first pattern on the car stays on the screen (right box in (b)) and the car moves behind a screen with an iconic representation of its operation. Children are then asked to predict the outcome with a new set of four choices on the left (b). In training trials (b), children answer until they are correct and then see the screen lift to reveal the car with the new pattern (c). The critical response in test trials is shown in (d), with the car occluded after passing behind two screens. No feedback is provided on these trials.

In the experiment, preschoolers were shown displays on a touchscreen monitor in which a car with a colored pattern appeared. Cars had one of two patterns (dots/stars) occurring in one of two colors (red/blue). A car with one choice of these features drove on-screen and children were required to select which pattern it matched from a menu with all four possible options (Fig 1a). This ensured that they attended to the features of the cars and knew them at the start of each trial. For this response, children were required to respond until they answered correctly, with incorrect responses penalized by a buzzing noise and correct responses rewarded by a trumpet sound. The car then drove behind a single screen, making its pattern (but not wheels) occluded (Fig 1b). It jiggled behind the screen to indicate that a transformation was taking place. Children then responded with what they expected the car to look like when the screen lifted.

The experiment began with 6 explicit training trials in which children were provided with verbal feedback and instruction on the operation provided by each of the screens. Participants then entered an second training phase in which they received no verbal feedback from the experimenter, but were required to answer single-screen trials until correct, at which time the screen lifted to reveal the correct outcome (Fig 1c). Presentation of operations in this phase was in blocks of 6 single operations (red, blue, dots, stars, and two with no changes, in random order).

Children stayed in the training phase until they answered 5 out of 6 correct in a block. After meeting this criterion, the experiment progressed to a test phase. Here, children were shown blocks containing 2 single screen displays with feedback, and 4 two-screen displays *without* feedback (Fig 1d). In these critical trials, children observed the car pass behind one screen, then the next, without seeing it in between. As in training, they were required to answer correctly to the first question on the car’s initial pattern, but crucially received *no* feedback on their selected outcome after the car had passed behind both screens. The screens never lifted to reveal the car after selection and the experiment simply progressed to the next trial.

Screens were chosen to be iconic of the color and pattern transformations because we are primarily interested in how children combine these operations *when they know them*, not how hard it is for children to learn each individual function. However, sometimes the screen would not cause a change—for instance, if a car with blue dots drove behind a blue screen. These trials were included in order to ensure that children really paid attention to the pattern on the screens and did not just “flip” the relevant dimension. We refer to transformations in which a feature changed (e.g. red car behind blue screen) as *change (CH)* screens, and transformations which did not change a feature (e.g. blue car behind blue screen) as *identity (ID)* screens.

Children were run in the experiment for a maximum of approximately 30 minutes, and were shown a short “reward” animation every three responses in order to keep the experiment interesting. Stimuli were presented using Kelpy, the Kid Experimental Library in Python, which is available under the GNU Public License from the first author [35]. This study was approved by the University of Rochester Research Subjects Review Board. Written informed consent was gathered from parents/guardians of the children involved.

### 2.1 Participants

Twenty-one children (10 females and 11 males) aged approximately 3.5–4.5 years (mean: 50.9 months; range: 42.9 to 53.9 months) were recruited to the Rochester Baby Lab. In order to assess overall levels of performance, all subjects were included in the following analysis, although two did not progress out of training.

## 3 Results

**Fig 2:**
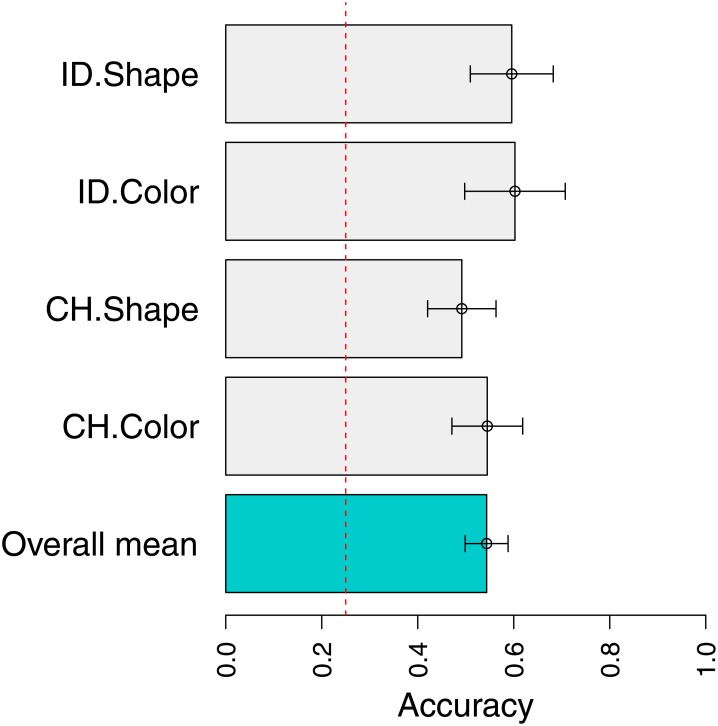
Mean response accuracies to single screens which performed change (CH) or identity (ID) operations. A chance rate of 25% is shown by the dotted red line.

Fig 2 shows mean first response accuracies to single screen trials, broken down by each type of function: change (CH) color, change pattern, identity (ID) color, identity pattern. Means and standard errors were computed using a mixed effect logistic regression, taking into account subject intercepts and slopes by function type [36]. This type of analysis is well-suited to unbalanced designs where children have been run for varying amounts of test and training items. The red line corresponds to a chance rate of 25%, for guessing at random from the four possible choices. This figure shows that in each condition children are substantially above chance on single screen displays. They thus learned the operations performed by each iconic screen. However, children are not very good at this task—their overall accuracy is near 50%. This likely results from the fact that the task is somewhat complex, requiring tracking of multiple values of multiple dimensions.

**Fig 3:**
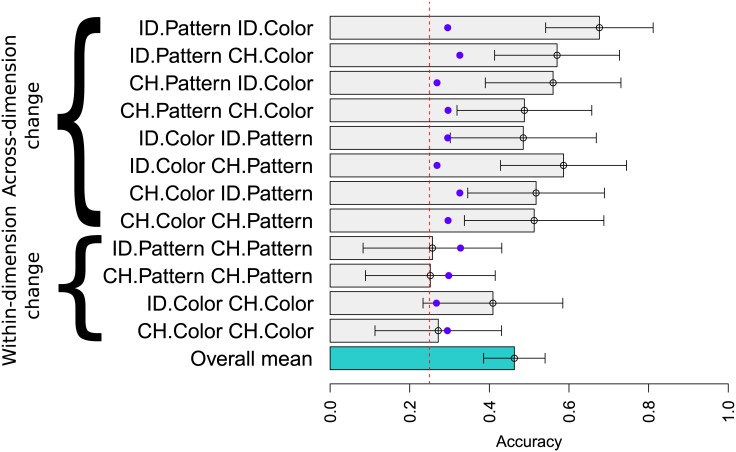
Mean response accuracies to double screens, each of which performed change (CH) or identity (ID) operations. Error bars show confidence intervals. A chance rate of 25% is shown by the dotted red line. The blue dots correspond to the accuracy predicted under independent application of the single function accuracies in Fig 2.

Critical two-screen test trials are shown in Fig 3. Again, error bars were computed via a mixed effect logistic regression with subject intercepts by function type (a model with random slopes did not converge). First, this figure shows that the overall mean response (teal bar) is substantially higher than the chance rate of 25%. The overall accuracy is also only slightly worse than the overall accuracy on single screen displays, meaning that knowledge of compositions is available to learners after training on single screens despite the fact that feedback was never provided on these two-screen trials.

The other bars in this plot show performance broken down by the type of function performed by each of the two screens. Despite above chance performance overall, children are at chance in conditions involving feature changes within the same dimension (e.g. CH-Color/CH-Color). In such a condition, a red circle car would go behind a blue screen (changing its color) and then behind a red screen (changing its color again). This requires keeping track of several different values within the same dimension, and—because the features are binary—realizing that a feature changes and then reverts back to its original value. Note that children are not just having difficulty in this condition in tracking the order in which the functions applied: that would predict 50% performance in these conditions (since children would have the other dimension right). Instead, it appears that children’s performance breaks down completely in this case, not unlike total breakdowns seen in object tracking [32–34].

These detailed patterns of responses also rule out several other hypotheses about children’s performance. First, it is clear that children are not above chance by merely applying one of the two screens. Such a tactic would predict 0% accuracy on conditions with two changes (e.g. CH-Color-CH-Pattern and CH-Pattern-CH-Shape) since application of only one screen would *always* give the wrong answer. Instead, children performed particularly well in these two conditions, with a mean accuracy of nearly 50%, trending above even the overall experiment mean. Responses in these conditions are each individually significantly above the 25% chance level (CH-Color-CH-Pattern accuracy is 50%, *p* = 0.003 when compared to chance; CH-Pattern-CH-Color is 55%, *p* = 0.002 when compared to chance). Children’s success in these conditions provides strong evidence that they do not simply choose one box to apply.

Moreover, children’s performance surpasses their expected performance based on their ability to apply single screens. The blue dots in Fig 3 show the performance that would be expected if children’s accuracy was determined by independent application of each function, with individual function accuracies determined by the performance on single boxes. Thus, the blue dot for CH-Color/CH-Pattern corresponds to the probability of success on a CH-Color operation followed by the probability of success on a CH-Pattern operation, according to Fig 2. Because the accuracies in Fig 2 are near 50% for each function type, application of two of these correctly is near 25%(= 0.5 ⋅ 0.5). Across nearly all function types other than changes within the same dimension, children are substantially above this performance level. This suggests that their failures are not due to independent failures on each screen. This pattern could occur if, for instance, children’s low accuracy in Fig 2 was driven by not paying attention on all trials, rather than not knowing the right answer.

**Fig 4:**
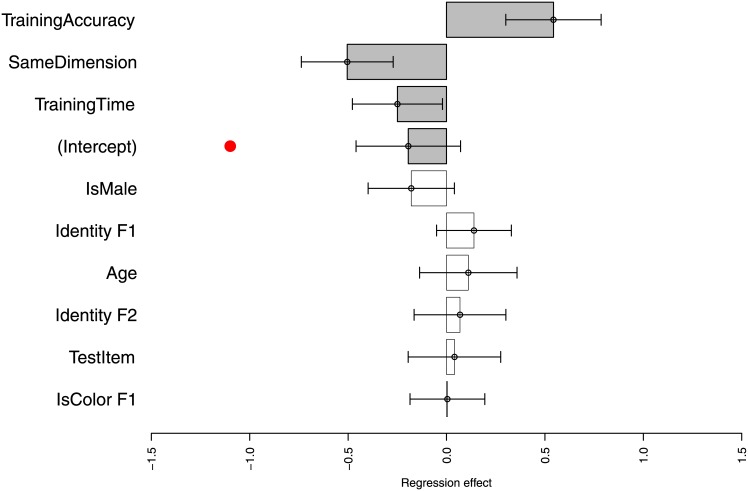
Coefficients in a mixed-effect logistic regression predicting accuracy on test (two-screen) trials from a number of predictors. There intercept here, representing the mean accuracy, should be compared to the chance rate of 1/4 (red dot, at *logit*(0.25) on this scale), the overall chance guessing rate for 4 options. All other coefficients should be compared to the *x* = 0 line, showing whether they had a statistically significant influence on response accuracy. Effects significant at *p* < 0.05) are shown with gray bars.

Responses were also analyzed using a mixed-effect logistic regression [36] with intercepts by subject (a model with random slopes did not converge). This analysis lets us simultaneously evaluate the influence of multiple factors of children’s response accuracies, and determine their mean accuracy while controlling these factors. Fig 4 shows estimated coefficients and standard errors. The coefficients here correspond to whether the first function is an identity function (Identity F1), the second is an identity (Identity F2; this is computed as whether the second function is an identity function on the *output* of the first function), the first change is a color dimension (IsColor F1), the second is the same feature dimension as the first (SameDimension), children’s age, whether the child is male, the child’s training accuracy, the number of items it took the child to progress on to testing, and the number of test items seen so far. The binary predictors here (Identity F1, Identity F2, IsColor F1, SameDimension, IsMale) are all negative sum coded and the continuous predictors are all standardized, meaning that the intercept can be interpreted as the mean response accuracy across all predictors. In this figure, coefficients far from the zero line indicate significant influences on response accuracy. This shows that there are three significant predictors: children are substantially worse when both boxes operate on the same dimension (SameDimension) (*β* = −0.50,*z* = −4.23,*p* < 0.001). This means that when the first box changes a color, children are worse when the second box also changes a color, and analogously when both boxes change pattern. Children are worse when they take longer to reach testing (*β* = −0.25, *z* = 2.14, *p* = 0.03). Children with better training accuracy also perform better on testing (*β* = 0.54, *z* = 4.40, *p* < 0.001). Importantly, the intercept here represents the mean accuracy controlling for all other effects. This shows that the intercept (*β* = −0.19) is significantly higher than the chance rate *logit*(0.25) shown by the red dot in the graph (*p* < 0.001), indicating that children are substantially above the chance guessing rate. They are not, however, significantly different from 50% accuracy (*p* = 0.15). These results revealed no significant effect of age, indicating that we have no evidence older children are better at this task. This might suggest that such compositional reasoning is attained earlier than about 3.5 years of age so that children in the range 3.5 ∼ 4.5 are not differentially capable of dealing with composition.

We additionally analyzed the pattern of errors children made in their responses. To do this, we looked only at incorrect responses and tried to predict which components of an incorrect response children were likely to get correct. In a mixed effect regression, we found that these responses were substantially more likely to provide a response with the *second* screen correctly applied rather than the first (*β* = 1.15, *z* = 4.946, *p* < 0.001). This effect was independent of whether the first and second screens were colors or not (|*β*|<0.1, *z* < 0.5, *p* > 0.65). This suggests that children’s difficulty with composition may be in tracking or encoding the features of the car and the first screen while focusing on the second, temporally more recent, screen.

## 4 Discussion

These results provide evidence that preschoolers spontaneously infer the outcome of combinations of function applications after receiving training on only the individual pieces. Their performance on two screen displays—though not perfect—is comparable to their performance on single screen displays. These results suggest that knowledge of composition does not require training for preschoolers beyond learning the individual pieces: children at this age have already learned how to predictively combine operations. This does not mean that children have never required instruction (or data) for learning about composition, but it does mean that whatever they have learned before the experiment began is abstract enough to apply to novel functions.

It is worth noting that while our experiments were motivated by compositionality, there are likely *non*-compositional models that could capture these results. For instance, children may learn that each screen constrains the possible outcomes, and combine constraints in order to predict the outcome. The challenge for these theories would be to state combination in a way that cannot be viewed (or perhaps cannot be viewed naturally) as function composition. There are also challenging data points for such theories even in this simple experiment: for instance, they would have a difficult time explaining why children are *below* 50% on within-dimension CH/CH trials, a fact that almost certainly depends on the details of how constraints may be combined. Stronger results could likely be provided by displays with more features and operations in order to fully probe the extent of children’s ability and distinguish alternative models. We therefore take our results as a first step that provides suggestive—not definitive—evidence for compositionality.

Our analysis revealed some subtle facts about which compositions are easy and difficult for young learners. Changes within the same feature dimension are difficult, but two changes across dimensions are as easy as one. The limitation within dimensions presents a challenge to the purest form of compositional theories: what type of mechanism could correctly compose, but only when the composed functions operate on separate feature dimensions? One theory is that setting a feature value in memory is slow. Thus, when the value of two features change rapidly, children may get confused about what the resulting value is. On the other hand, if one feature changes and then a different feature changes, both “write” processes can occur in parallel and not interfere with each other. Difficulties dealing with rapidly sequential updates to a variable are common in parallel programming in computer science, (resulting in a so-called *race condition*). This view is consistent with the pattern of children’s errors, where it appears that the transformation of the first screen is most likely to get overwritten or lost.

Interestingly, however, children’s performance is also *below* 50%, indicating that their failure is perhaps more than a problem with remembering both screens. It is more likely to be a complete breakdown, because forgetting a single screen would still give 50% performance in the two-screen trials (since half the time, one will be an identity screen). This may indicate that memory mechanisms are fundamentally incapable of tracking multiple updates to the same feature dimension, perhaps because two rapid updates interfere in a particularly destructive way.

In the introduction, we discussed two forms of compositionality corresponding to whether learners explicitly represent a combination of functions *f*_2_ ∘ *f*_1_ or whether they merely have the capacity to successively apply them to a representation *f*_2_(*f*_1_(*x*)). The present experiment does not strongly distinguish between these possibilities; it is possible that children only track the object moving behind the screens, successively updating its visual features (corresponding to *f*_2_(*f*_1_(*x*))). It is also possible that children could explicitly know that two screens together can be chunked into a single unit, the function *f*_2_ ∘ *f*_1_. These possibilities might be disentangled by future work where children’s ability to treat compositional functions as single units (e.g. in other compositions) could be evaluated.

In addition to demonstrating compositional ability, these results also suggest that children aged 3.5 ∼ 4.5 are able to represent *functions*, not itself a trivial capacity. In particular, to perform above chance children must be able to represent the fact that each of four screens performs a particular change to an object’s features, and that that change occurs even if the outcome is not directly observed. Such an ability to represent functions themselves might be viewed as an even more basic ability than compositionality. However, fluency with functions can only yield more complex computations if those functions can be combined to form novel combinations—which our results indicate preschoolers are likely able to do.

We consider this capacity for representing functions themselves as potentially a basic fact about the organization of cognitive computation. In modern computer systems, encapsulated functions play a critical role by allowing complex computations to efficiently and easily be built from simpler components. For instance, programs are constructed only out of simpler elementary operations (e.g. +, −, ⋅, /, *for*, *if*, etc.) which are eventually directly interpretable by the computer’s hardware. Using only these kinds of primitive functions, one can create a structure implementing a more complex computation, like *f*(*x*) = *x* ⋅ *x* ⋅ *x*+*x* − (*x*+1)/*x*. The capacity to combine such elementary operations in arbitrary ways is extremely powerful, and an ability to manipulate functions themselves potentially allows a huge range of computations to be executed using very little “built in” knowledge (ie. few primitives). Indeed, the simplest computational systems like lambda calculus [23, 25] and combinatory logic [24, 37] “build in” almost nothing, essentially *only* the rules of function composition. A striking result in formal logic holds that arbitrary (ie. Turing-complete) computational processes can then be built out of nothing more than this capacity for composition [23]. This means that the ability of children to represent, manipulate, and compose functions may point to how they are able to acquire operations of substantial computational complexity, while requiring only very simple cognitive machinery. In this sense, compositionality may be *the* key component of LOT theories that allows arbitrarily complex computations to be represented by learners.

Our study was in large part motivated by LOT learning models, but it is also interesting to consider how to interpret these results in the context of larger theoretical divides in cognitive science. It may be productive, for example, to determine how such learning might be captured in connectionist or parallel distribution processing [38] approaches: how might one capture learning of separate functions which can be composed, without requiring any additional training on composition? Is it possible to set up such a system in a way that cannot be interpreted as presupposing compositionality itself? Or, it may be that compositionality is basic enough that, as in LOT theories, it should be assumed as a core computational primitive.

## 5 Conclusion

These results are an early step in linking contemporary structured learning models with infant and early childhood experimental studies. An ability to combine novel functions appears to be robustly present by three or four years of age, with children requiring no explicit training on combination once they have learned individual functions. This suggests that learning theories based around assuming compositionality are behaviorally plausible and that a capacity for combining mental operations may be one of the mechanisms that supports children’s creation of rich conceptual systems.
